# Revealing of *Mycobacterium* *marinum* Transcriptome by RNA-seq

**DOI:** 10.1371/journal.pone.0075828

**Published:** 2013-09-30

**Authors:** Sen Wang, Xinran Dong, Yongqiang Zhu, Chuan Wang, Gang Sun, Tao Luo, Weidong Tian, Huajun Zheng, Qian Gao

**Affiliations:** 1 Key Laboratory of Medical Molecular Virology, Institutes of Biomedical Sciences and Institute of Medical Microbiology, Shanghai Medical College, Fudan University, Shanghai, P. R. China; 2 Institute of Biostatistics, School of Life Sciences, Fudan University, Shanghai, P. R. China; 3 Shanghai-MOST Key Laboratory of Health and Disease Genomics, Chinese National Human Genome Center at Shanghai, Shanghai, P. R. China; Cornell University, United States of America

## Abstract

Transcriptome analysis has played an essential role for revealing gene expression and the complexity of regulations at transcriptional level. RNA-seq is a powerful tool for transcriptome profiling, which uses deep-sequencing technologies to directly determine the cDNA sequence. Here, we utilized RNA-seq to explore the transcriptome of *Mycobacterium* *marinum* (*M. marinum*), which is a useful model to study the pathogenesis of *Mycobacterium tuberculosis* (*Mtb*). Two profiles of exponential and early stationary phase cultures were generated after a physical ribosome RNA removal step. We systematically described the transcriptome and analyzed the functions for the differentiated expressed genes between the two phases. Furthermore, we predicted 360 operons throughout the whole genome, and 13 out of 17 randomly selected operons were validated by qRT-PCR. In general, our study has primarily uncovered *M. marinum* transcriptome, which could help to gain a better understanding of the regulation system in *Mtb* that underlines disease pathogenesis.

## Introduction

*Mycobacterium tuberculosis* (*Mtb*) is a pathogen causing tuberculosis (TB), which leads to approximately 2 million deaths and 10 million new infections annually [1]. With the emergence of multidrug-resistant (MDR) and extensively drug-resistant (XDR) TB, the current situation is more challenging. In order to search for new antibiotics and more optimized treatment strategy against *Mtb*, a comprehensive understanding of the intracellular lifestyle of this organism is urgently needed [2,3]. *Mycobacterium* *marinum* (*M. marinum*), a pathogenic mycobacterium that causes disease in fish and amphibians, is genetically close to *Mtb* [4]. Recent studies by Tobin, D.M., et al. and other researchers demonstrated that *M. marinum* was a useful model to study the pathogenesis of TB, and furthermore, to explore potential therapeutic drug target [5-7].

Transcriptome analysis, which studies all the RNA transcripts during a particular physiological condition, has played a central role for detecting gene expression and transcriptional regulation. It includes investment of transcript structure, operon linkages and absolute abundance of transcripts. However, these information has not been determined on a genome-wide scale for any bacterium until 2009, Passalacqua, K.D., et al. reported the first single-nucleotide resolution view of *Bacillus anthracis* transcriptome [8]. Then a genome-wide map of *Helicobacter pylori* transcriptional start sites (TSSs) and operons were revealed by Sharma, C.M., et al. in 2010. Through transcriptome study, they discovered hundreds TSSs within operons and opposite to genes, which indicates the complexity of gene expression in *Helicobacter pylori* by uncoupling polycistrons and antisense transcription [9]. As for the mycobacterial transcriptome study, Arnvig, K.B., et al. revealed an extensive presence of non-coding RNA in *Mycobacterium tuberculosis*, indicating that post-transcriptional regulations might play an important role in bacterial adaptive responses to changing environments [10]. All these fundamental studies above applied RNA-seq technology, which has led to a fast development of transcriptome study for its relatively lower cost and much more precise measurement of transcripts comparing with hybridization-based microarray assays [11].

RNA-seq is an excellent approach for transcriptome profiling, which uses deep-sequencing technologies to directly determine the cDNA sequence. Besides its relatively lower price and precision mentioned above, RNA-seq is suited to the application of detecting transcripts which correspond to existing genomic sequence. Vera JC, et al have used RNA-seq to reveal the transcriptome of the Glanville fritillary butterfly, which makes RNA-seq especially attractive for non-model organisms with genomic sequences not known [12]. RNA-seq can also reveal the precise location of transcription boundaries to a single-base resolution and have a relatively low background comparing with microarray [11]. Comparison between the two techniques has been reported in several species such as *Candida* *parapsilolis* [13], *Candida albicans* [14] and *Caenorhabditis elegans* [15]. It has also been applied to discover non-coding RNA at genome wide range [10,16]. All factors above make RNA-seq useful for studying complex transcriptomes. However, the transcriptome of *M. marinum* has not been revealed experimentally up to now although current tools and database (http://mycobrowser.epfl.ch/marinolist.html) have already enabled us to scan its genome and transcriptome from *in silico* prediction [17].

In this research, we took advantage of RNA-seq technology to study the transcriptome of *M. marinum* in exponential and early stationary phase cultures, and investigated the functions for genes that expressed differently in these two phases. To compare the expression levels of different genes, data for CDSs were presented in the form of reads per kilobase per million reads (RPKM). In addition, we predicted potential operons and used qRT-PCR for validation.

## Results

### High quality RNA preparation and sequencing profile

#### Ribosome RNA (rRNA) removal before cDNA library construction

**Figure 1:**
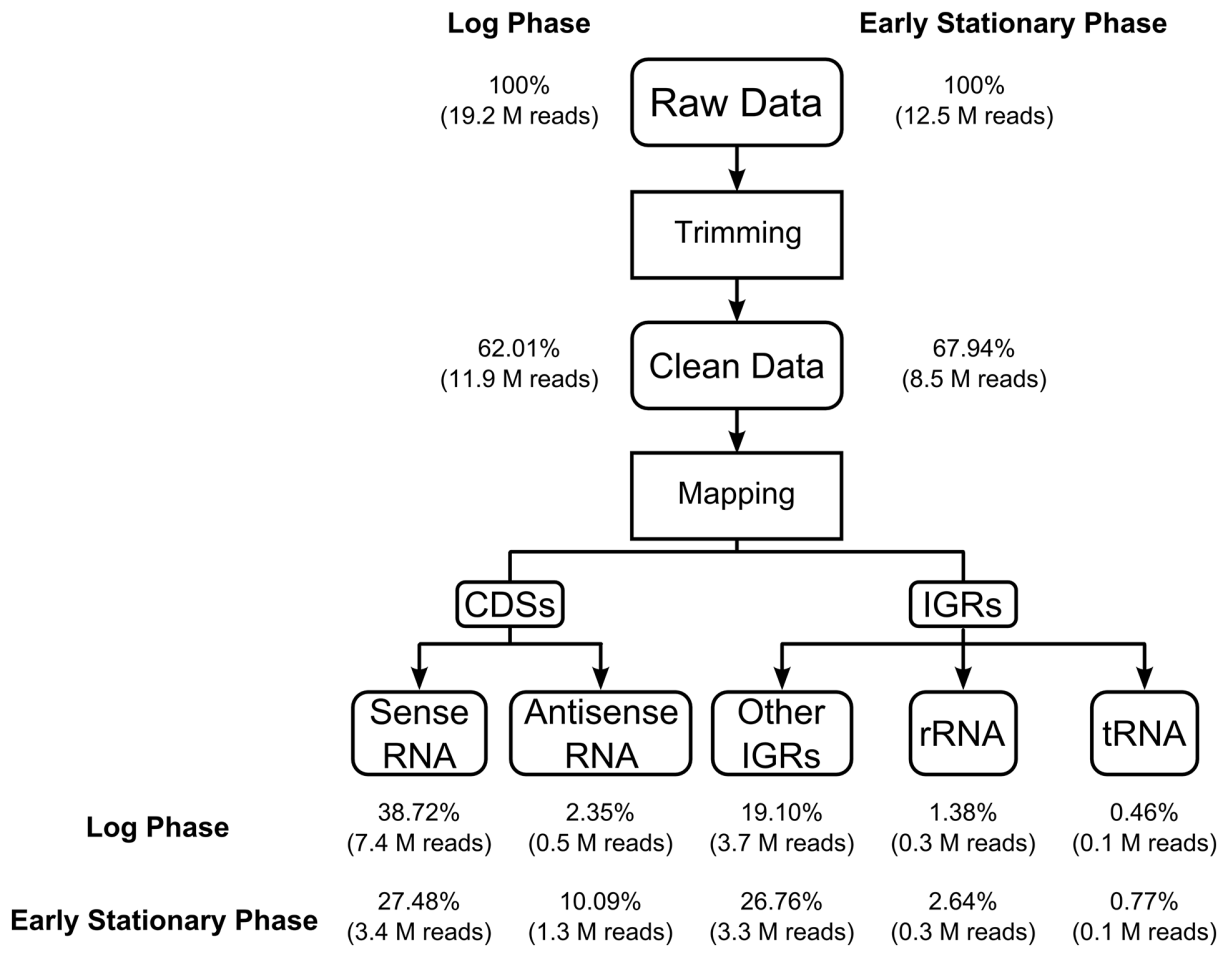
RNA sequencing profiles for both exponential and early stationary phase cultures of *M. marinum*. Number of reads obtained from data processing/mapping results and their percentage were indicated for both exponential and early stationary phase cultures.

Removal of signals from rRNA is a vital step of RNA-seq technology because these signals may reduce the coverage of mapped results, while decreasing the sequencing meantime. There is only one set of rRNA genes throughout the whole genome of *M. marinum* while they account for 85% of total RNA according to our previous trial (data not shown). In terms of this, we applied an rRNA removal step before cDNA library construction. As a result, 0.3 million reads mapped to rRNA were left in either log phase or early stationary phase culture, which account for 1.38% and 2.64% of total sequenced reads separately (Figure 1, Table S1). All 5452 genes were detected within two million uniquely mapped reads in log phase sample. While in the early stationary phase sample, one million uniquely mapped reads could cover all genes in the genome (Figure S1 in File S1). A biological replicate of log phase culture was set to evaluate the reproducibility of gene expression profiles using RNA-seq technology. The Spearman correlation coefficient between two samples (r=0.867) indicates the overall pattern of relative gene expression appears quite similar between two biological replicates (Figure S2 in File S1).

#### The genome-wide distribution of sequenced reads

In order to investigate the transcriptome of *M. marinum* by RNA seq, we harvest bacteria cultures at OD600=0.8 (log phase) and at 4.0 (early stationary phase). RNA was extracted initially, and then followed by the rRNA removal step described above to generate the cDNA library, which was analyzed by Illumina-based sequencing eventually.

Two transcriptome files, a log phase of 19.2 million reads and an early stationary phase culture of 12.5 million reads, were generated in total. Adapters of all reads were removed before next step. The generated reads have an average length of 100bp and the sequencing quality of all reads was shown in Figure S3 in File S1. Reads with sequencing error or low quality value (more than 50% of all bases in a single read have quality value <5) were filtered and the rests were defined as clean data. Respectively, 11.9 million and 8.5 million clean reads were generated from two phases’ transcriptome files, accounting for 62.01% and 67.94% of their total reads. The result of reads mapping was shown in Figure 1.

### The transcriptome of *M. marinum*

#### Gene expression profile analysis

**Figure 2:**
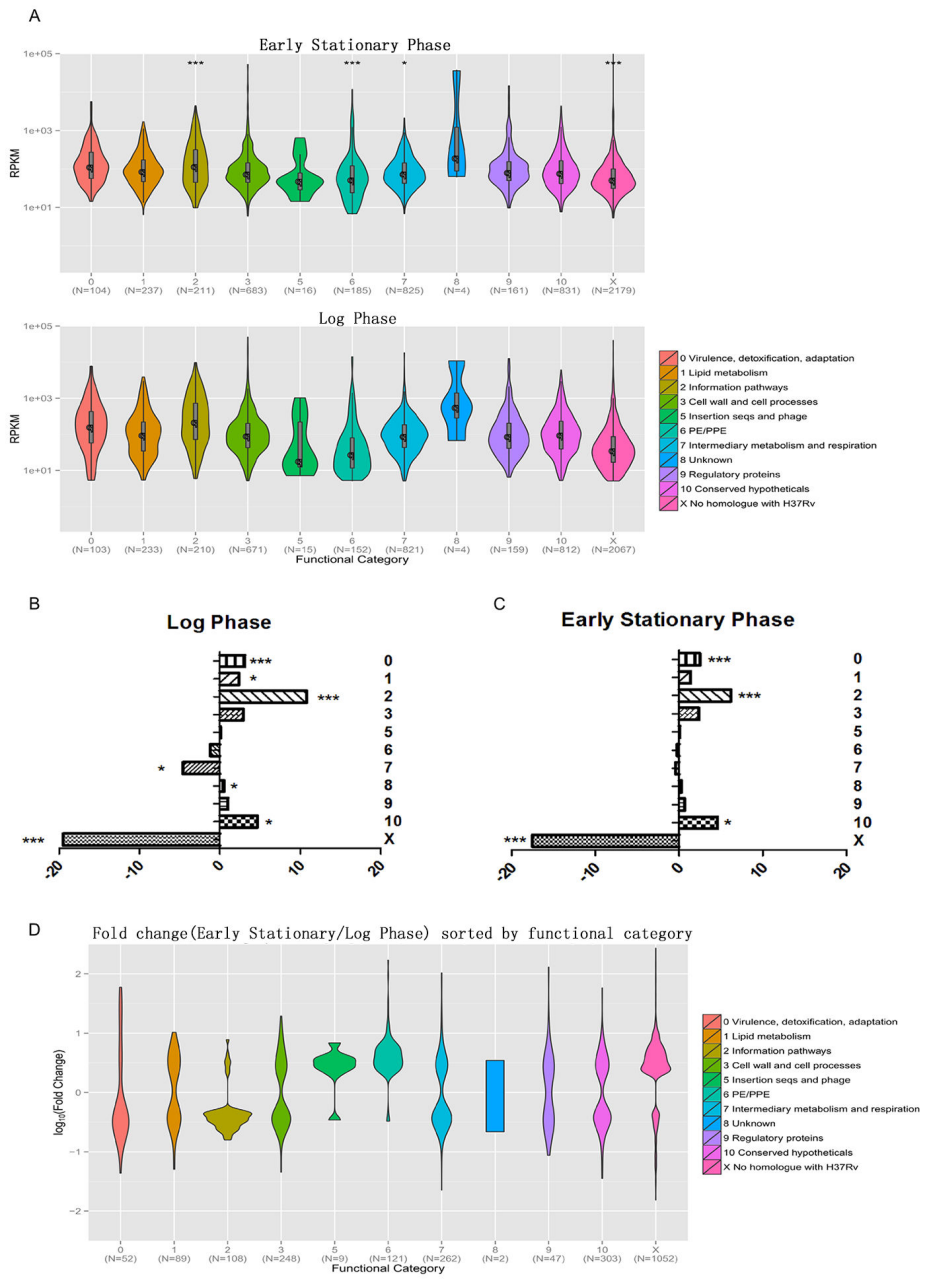
Representation of functional categories in log- and early stationary phase transcriptome. (A) Genes of *M.m* were grouped according to their homologs in *M.tb*. Numbers on the x-axis indicates 11 categories and gene numbers contained in each category. Numbers on the y-axis represents RPKM values. RPKM values of each category were analyzed between two samples using Wilcoxon test. * p<0.05, *** p<0.001. (B and C) Values on the x-axis indicate the difference in percentage; positive values indicate over-representation of a particular category compared to the percentage observed in the annotated genome whereas negative values represent under-representation. (B) Difference in percentage of selected categories of top 10% log phase transcripts (n=525, Fisher’s exact test). (C) Difference in percentage of selected categories of top 10% early stationary phase transcripts (n=544, Fisher’s exact test). (D) Genes with significant changes (fold change>2, q value<0.001) were grouped.

To study the gene expression profile of *M. marinum*, we focused on CDSs with RPKM≥5 in either sense or antisense direction [10] (Table S2). There were 5245 genes with RPKM≥5 during log phase culture, while in early stationary phase culture, the number is 5434. Among these genes whose RPKM≥5 between two samples, 5240 were shared and 5 genes merely expressed in log phase, while 194 genes were specific during early stationary phase (Table S3). All CDSs were grouped according to the functional classes of their homologs in *Mtb* [18]. 2184 CDSs have no homologs in *Mtb*, which accounts for 40.1% of all annotated CDSs in *M. marinum*. The rest CDSs were classified into 10 categories (http://genolist.pasteur.fr/TubercuList/help/classif-search.html). To emphasize, in our results there were no “category 4” which stands for “stable RNAs”. Figure 2A shows distribution of RPKM along with the number of genes in each category of two samples. The median RPKM of each category was shown in Table S4. Then we analyzed the RPKM distribution of each category between two samples using Wilcoxon test. “Information pathways” (category 2) were significantly up regulated (p<0.001) in log phase culture as well as “Intermediary metabolism and respiration” (category 7, p<0.05). In early stationary phase culture, “PE/PPE family” (category 6) and category X (No homologue with H37Rv) were significantly up regulated (p<0.001) comparing with those in log phase culture. To verify the results, we randomly selected 24 genes (10 from up- regulated genes, 10 from down-regulated genes and 4 from unchanged genes) to perform qRT-PCR experiment (Table S5). For the 10 genes which were down-regulated in RNA-seq data, eight of them were confirmed by qRT-PCR. However, only half of the up-regulated genes (five out of ten genes) could be verified by qRT-PCR, while the rest of genes belonging to this category did not have significant changes. The qRT-PCR results of unchanged genes were in accordance with the results observed in our RNA-seq data.

#### Association between expression level and gene function

To test for the association between expression level and gene function, 10% of CDSs with the highest RPKM from both samples were selected and studied according to their function distribution as described above. By comparing the frequency of each category across the whole genome, we found that category 0 (Virulence, detoxification, adaptation), and 2 (Information pathways) were significantly over-represented (P-value<0.0001, Fisher’s exact test). This shares a same tendency with category 1 (Lipid metabolism), 8 (Unknown), and 10 (Conserved hypotheticals) (P-value<0.05) (Figure 2B), which is consistent with what is expected for log phase culture. The actively growing bacteria over-represented mRNA transcripts encoding proteins involved in “lipid metabolism” and “information pathways”. Category X (No homologue with H37Rv) (P-value<0.0001) and transcripts belonging to “intermediary metabolism and respiration” (P-value<0.05) were under-represented in log phase culture. The early stationary phase culture showed similar results with category 0, 2 (P-value<0.0001) and 10 (P-value<0.05) (Figure 2C) being over-represented. These results were also reflected in Table 1, where we showed 30 CDSs with highest RPKM and the corresponding functional classes. For both log and early stationary phase culture, almost one-third of highest RPKM belong to the category “cell wall and cell processes”. Meanwhile, there were also representatives of “information pathways” and “PE/PPE family”. We also compared our results with previous transciptome study in *Mtb* by Arnvig, K.B., et al [10]. RPKM of *M. marinum* genes and their homologues in H37Rv were compared, and spearman correlation coefficients were 0.495 and 0.326 for log phase and early stationary phase separately (Figure S4 in File S1). Then we further analyzed correlation coefficient of each functional category (Figure S5 in File S1). For the log phase culture, there were four categories with spearman correlation coefficient above 0.5 (0 Virulence, detoxification, adaptation; 2 Information pathways; 3 Cell wall and cell processes; 8 Unknown), while for the early stationary phase culture, spearman correlation coefficient of category 2 (Information pathways) and category 8 (Unknown) were above 0.5.

**Table 1:** Ranking of the most abundant coding transcripts in log and early stationary phase.

	name	length	Category	RPKM
Log phase				
	esxB	303	3	50652.89
	MMAR_5556	315	X	40854.49
	esxA	288	3	26542.65
	esxP_2	297	3	20071.38
	fdxA_2	345	7	18528.19
	acpM	348	X	15257.81
	MMAR_4786	300	6	14794.73
	esxP	297	3	13370.97
	whiB1	255	9	12950.39
	MMAR_3655	318	8	10954.16
	rpmC	243	2	10034.55
	MMAR_5447	297	6	9540.277
	rpmJ	114	2	8349.4
	cspA_1	204	0	8046.823
	esxN_1	285	3	7653.413
	cspA	204	0	7647.709
	whiB4	351	9	6735.596
	MMAR_1426	411	X	6444.57
	MMAR_0966	210	10	6250.998
	rpsG	471	2	5777.113
	MMAR_5437	288	9	5673.893
	rpmG2	168	X	5395.081
	MMAR_0724	333	X	5349.003
	atpE	246	7	5313.055
	esxB_1	303	3	4960.48
	MMAR_4277	333	3	4670.253
	esxN_3	285	3	4649.54
	MMAR_4306	156	X	4612.031
	MMAR_1457	231	X	4462.532
	MMAR_5440	312	10	4193.604
Early stationary phase				
	MMAR_5556	315	X	431455.2
	esxB	303	3	54927.45
	esxP_2	297	3	45801.19
	MMAR_3655	318	8	38003.32
	esxP	297	3	23859.81
	glnB	339	9	15081.94
	esxA	288	3	13469.56
	esxM	297	3	12781.58
	MMAR_4786	300	6	12211.53
	whiB4	351	3	7220.467
	MMAR_5447	297	9	6391.867
	esxN_3	285	6	5895.651
	cspA_1	204	3	5792.304
	acpM	348	0	5277.71
	rpmJ	114	X	4535.116
	MMAR_3248	357	2	4488.187
	esxN	285	10	4032.88
	MMAR_4277	333	3	3946.844
	MMAR_4167	312	3	3796.862
	MMAR_1893	381	10	3760.808
	MMAR_2878	417	10	3726.62
	MMAR_1457	231	X	3723.4
	MMAR_2879	195	X	3673.473
	whiB1	255	X	3644.408
	esxB_1	303	9	3581.075
	MMAR_2706	495	3	3222.692
	MMAR_2905	261	10	3211.932
	MMAR_4784	915	X	3154.366
	MMAR_1868	975	X	3115.166
	cyp278A1	1284	X	3046.001

* 0 Virulence, detoxification, adaptation; 1 Lipid metabolism; 2 Information pathways; 3 Cell wall and cell processes; 5 Insertion seqs and phage; 6 PE/PPE; 7 Intermediary metabolism and respiration; 8 Unknown; 9 Regulatory proteins; 10 Conserved hypotheticals; X No homologue with H37Rv

#### Significant gene expression changes

To further explore the two gene expression profiles, we looked into those genes with significant changes between two samples. Genes with at least 2-fold change and FDR (false discovery rate) less than 0.001 were defined as differentially expressed genes (See Materials and Methods). Totally, 1446 genes were up-regulated in early stationary phase culture and 847 genes were down-regulated compared with log phase culture. Then these up and down regulated genes were grouped according to their functional classes as described above (Figure 2D). Among those genes belonging to “information pathways”, 95 were down regulated, which accounts for 88.0% of all 108 changed genes (Table S6). In early stationary phase, genes of “PE/PPE family” with significant changes were dominantly up regulated (116/121). Meantime, 262 genes belonging to “intermediary metabolism and respiration” changed at least 2-fold, among which 106 were up regulated and 156 were down regulated. For these genes with no homologues in *Mtb*, 1052 have significant changes between two samples and mostly (82.8%) were up regulated. Furthermore, 8 out of 9 genes were up regulated for the category of “insertion seqs and phage”. In contrast, when we refer to the category of “virulence, detoxification and adaptation”, more genes turn out to be down-regulated. Besides, genes encoding early secreted antigenic target of 6 kDa (ESAT-6) and culture filtrate protein of 10 (CFP-10) stayed unchanged between two samples, together with most of the key components forming ESX-1 secretion system (Table S7). Three genes (*MMAR_5439, MMAR_5368 and MMAR_1553*) encoding proteins known to be secreted by ESX-1 were down regulated in early stationary culture [19,20]. In addition, genes constituting ESX-5 secretion system were generally up regulated in early stationary phase culture together with a gene known to encode ESX-5 dependent secreting protein MMAR-3728.

### Operons prediction and validation by qRT-PCR

Operons are direct structures to determine whether genes next to each other are transcribed together, so we predicted operons on a genome-wide scale. We considered gene expression level in our sequence profile, coverage of intergenic regions (IGRs) and gene orientation as basic principles for operon prediction (See Materials and Methods). As a result, 898 operons were predicted in total throughout *M. marinum* genome from log phase culture and 978 from early stationary phase culture. In addition, there were 360 predicted operons in common between these two samples (Table S8). In both samples, most predicted operons were small transcriptional units containing only 2 or 3 genes. For example, in log phase culture, 860 operons have two or three genes, accounting for 96% of the total operons predicted (76% and 20% separately). In addition, 38 operons, almost 4% of all, contain 4 genes or more in our predicted results. The biggest operon predicted in log phase culture, *MMAR_1772*--*MMAR_1777*, includes six genes. These six genes encode five *pps* (polyketide synthase) family (*ppsA-ppsE*) and *fadD26*, the former of which participates in lipid metabolism while the latter has a potential role in activation substrates for the pps polyketide synthase.

**Figure 3:**
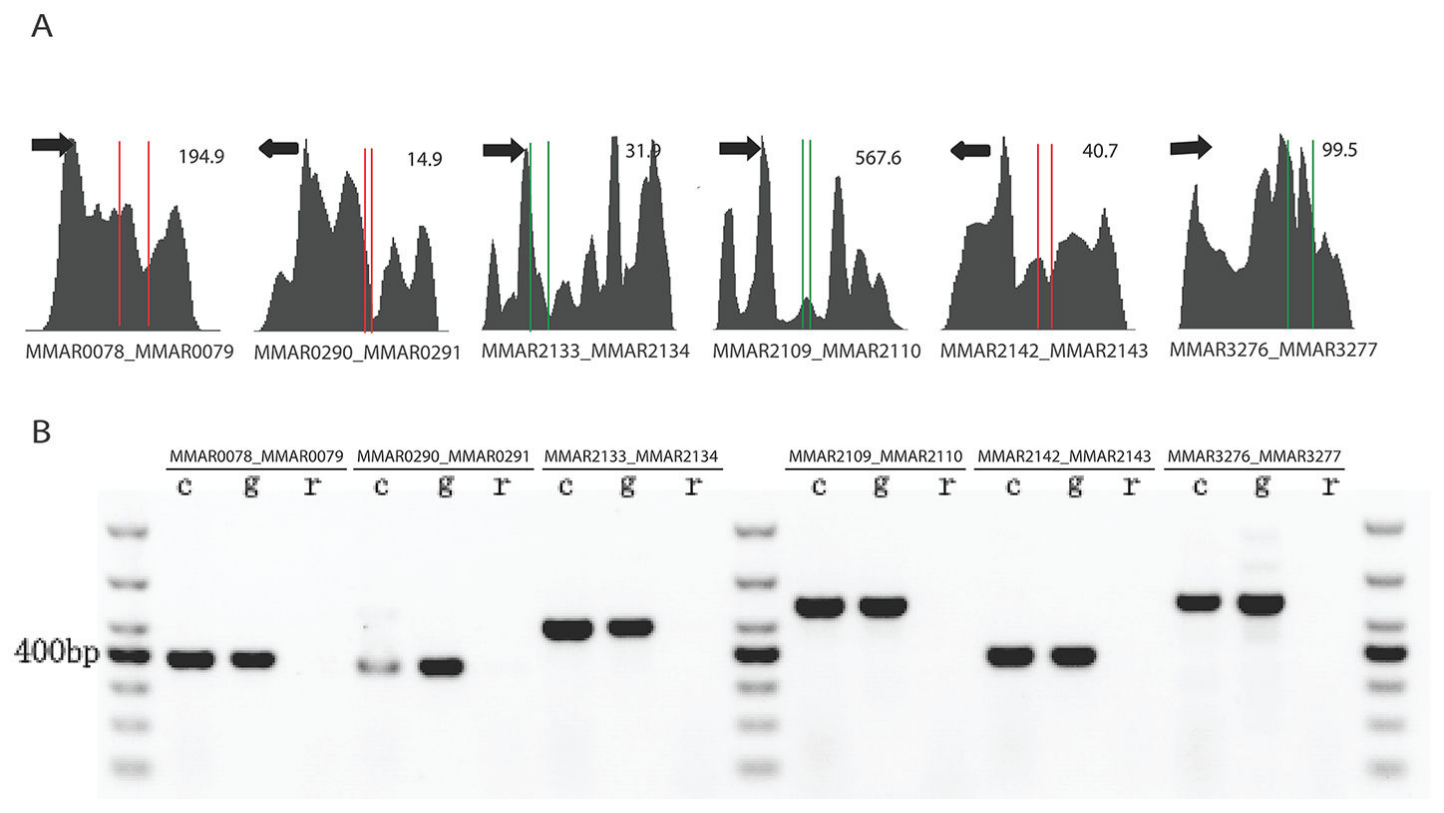
Sequencing traces of select putative operons and RT-PCR results. (A) Sequencing data of amplified regions. Numbers on upper right corner indicate the highest coverage of selected regions. Red lines indicate gene boundaries, green lines shows overlapped regions of two genes. Black arrows indicate direction of transcription. (B) Products of RT-PCR for 6 co-operonic gene pairs (1% agarose gel). c=cDNA template. g=genomic DNA template. r=RNA template.

Then we randomly selected 17 operons from log phase culture for validation using qRT-PCR. Of the 17 operons selected, 14 operons contain two genes and 3 operons contain three genes. Primers were designed to amplify the intergenic region between two genes using cDNA. In the case of operons having three genes, region between the first and the last gene was amplified (Figure S6 in File S1). Figure 3A shows six representative operons and their expression profile. And qRT-PCR results were shown in Figure S7 in File S1, from which we could find that 13 out of 17 selected operons were confirmed positive. Among these 13 operons, 11 operons have two genes and 2 operons have three genes. Figure 3B shows representative qRT-PCR results corresponding to predicted operon maps in Figure 3A.

## Discussion

Removal of signals from rRNA and high quality RNA preparation are crucial steps of RNA-seq technology. *M. marinum* genome has three rRNA copies, which encodes around 85% of total RNA content (data not shown). This number is close to previously reported 82% by Haas, B.J., et al., when they evaluated the effect of rRNA depletion on RNA-seq transcriptome profiles of *E. coli* [21]. Such high abundance of rRNA not only reduces the coverage of mapped results, but also decreases the sequencing depth when initial number of sequenced reads is set. To overcome this obstacle, a physical removal step has been applied before sequencing [22,23]. In this study, we used a newly developed Kit designed specifically for Gram-positive Bacteria to get rid of rRNA. As a result, less than 3% of reads were mapped to rRNA. Saturation curves were drawn to evaluate the coverage of CDSs within certain amount of reads using Number of genes detected against Number of uniquely mapped reads. Within two million uniquely mapped reads, all genes were detected in log phase sample. And the number of reads needed was one million to cover all genes for early stationary phase culture. These results benefited from the rRNA removal step before sequencing.

Gene expression profile as well as the association between expression level and gene function of *M. marinum* was investigated using RNA-seq in our study. PE/PPE family represents 9.1% of the coding capacity of *M. marinum*, compared with 7.1% for *Mtb* [4]. And it has been proposed that genes belong to PE/PPE family coevolved with the ESX loci, they underwent a specific expansion in the common progenitor of *M. marinum*, *M. tuberculosis*, and *M* *. ulcerans* [24]. Previous researches showed that different PE/PPE genes are expressed when the bacilli encounter environmental changes such as adaptation to stationary phase, deprivation of oxygen, encountering macrophages [25,26]. Our result showed 121 genes of PE/PPE family had significant changes and most of them (116) were up regulated in early stationary phase culture. And these significantly changed genes played important roles in the RPKM distribution differences between two samples in Figure 2A. It’s also of interest to look into the ESX-1 and ESX-5 secretion systems, which belong to type-VII secretion systems (T7SSs) in mycobacteria [27]. *MMAR_3728*, a PE_PGRS protein encodes gene that was proved to be ESX-5 dependent, was up-regulated in early stationary phase culture. This could correspond to the fact that genes encoding ESX-5 components were also generally up-regulated. Considering that 116 out of 121 genes with significant changes belong to PE/PPE family were up-regulated, our result strengthens the hypothesis that ESX-5 is a specialized protein secretion system that is devoted to the transport of PE/PPE family proteins [28]. We also found reads mapped in the antisense orientation or at IGRs accounts for 23.29% of total transcriptome in the exponential phase *M. marinum*, similar to the 28% reported for *M. tuberculosis* [10] and 27% reported for *Helicobacter pylori* [9]. While in the early stationary phase culture, these reads represent 40.26% of the total transcriptome, accounting for 1.7 fold (23.29%) of the exponential phase. This is possibly due to a tighter regulation of gene expression at a post-transcriptional level in early stationary phase culture, which may have a significant role in the response to stimulation of stress population heterogeneity [29]. Interestingly, there is an unusual highly expressed gene (*MMAR_5556*) whose RPKM is almost ten times higher than the rest. By blast this gene against reference strain (H37Rv) of *Mycobacterium tuberculosis*. It turns out to be homologue (86% identical sites) with MTS2823, which is small RNA (sRNA) as previously reported by Arnvig, et al [10]. They found that MTS2823 was the most abundant sRNA during exponential growth with a further more than six-fold increase during stationary phase. The expression values of *MMAR_5556* were in the same fashion here. This indicates that *MMAR_5556* may be misannotated as a CDS, and it is more likely to be an sRNA in *Mycobacterium* *marinum* . The expression profiles of log- and early stationary phases have been verified by qRT-PCR. However, not all changes in RNA-seq data could be confirmed by qRT-PCR, and the changes from qRT-PCR experiment were less significant compared with RNA-seq data in general. Besides the different treatments of RNA samples (an additional rRNA removal step for RNA-seq), the differences of gene expression results between these two approaches could be due to the variations between biological repeats, which is one of the limitations of this study. We also compared our RNA-seq results with former transcriptome study in *Mtb* by Arnvig, K.B., et al [10]. The correlation coefficients were found relatively low between these data sets using Spearman Test, this could be due to several reasons. First, the strategies for sample preparations are different. Arnvig, K.B., et al used tobacco acid pyrophosphatase to enrich for small transcripts before sequencing and chose to manually pick out reads mapped to rRNA. While in our study, rRNAs were physically removed before sequencing. This could lead to a general difference on the number of reads mapped to CDs, which varies the expression profiles in turn. Second, the time point for sample collection is different. Here we selected samples of log phase and early stationary phase to analyze transcriptome profiles of *M. marinum*, which could be distinguishable from the “stationary phase” they selected. This may explain the lower correlation coefficient for stationary culture. And again, the single culture analysis used in our study could be responsible for the bias as we discussed above.

Operon structure is important in prokaryotic genomes because it determines whether adjacent genes are transcribed together and this further implies whether genes in an operon are co-regulated. Thus, it’s of importance for operon prediction. RNA-seq technology has great advantages in operon prediction over hybridization-based microarray in aspects of supplying information of transcribed intergenic regions. Additionally, it also provides a far more precise measurement of levels of transcripts, as reviewed in 11,14. Price, M.N., et al. have established an operon prediction method for prokaryotes based on comparative genomic measures and distance between adjacent genes. The accuracy of their method turns out 85% and 83% for *E. coli* and *B. subtilis* separately [30]. Later, Passalacqua, K.D., et al. have successfully predicted operons in *Bacillus anthracis* using their own sequencing data file and 10 co-operonic gene pairs were validated by RT-PCR [8]. In the field of archaeal transcriptome study, Wurtzel, O, et al. defined more than 1000 operons in *Sulfolobus solfataricus* P2 using whole transcriptome sequencing approach [31]. TB Database offers the “Operon Brower” function to estimate whether two genes are transcribed together in *M. tuberculosis* H37Rv as well as syntenic gene order in related species based on expression correlation information collected from 1260 microarray assays (http://genome.tbdb.org/annotation/genome/tbdb/OperonBrowser.html). However, the information of transcribed IGRs is not available there and *M. marinum* is not included in the “Operon Brower” category mentioned above. So we applied a modified method based on transciptome analysis to identify the operons of *M. marinum*. Three hundred and sixty operons were predicted in common from both samples and 13 out of randomly selected 17 operons from log phase culture were confirmed by qRT-PCR. The accuracy is 76.5%, similar to the results in *E. coli* and *B. subtilis* mentioned above [30]. As for the 4 predicted operons that could not be validated here, it could be due to the limited sensitivity of current PCR system and certain genes may only be expressed under specific conditions. There are some known operons reported in *Mycobacterium tuberculosis*. As reviewed by Roback, et al, there are 26 verified operons, of which 22 have homologues in *M. marinum* [32]. We checked these 22 operons in our prediction results and found 18 (82%) of them could be confirmed (including partly matched because these reported operons could be incomplete according to the paper). The known mycobacterial operons missing in our results could either be due to their low expression in our sample or the differences of operon structures between *Mtb* and *M. marinum*. Besides, we also compared our prediction results from log phase sample with 761 predicted operons at MicrobesOnline (http://www.microbesonline.org/), and found 472 operons in common.

RNA-seq technology is also a powerful tool to search small RNA (sRNA) in bacteria. Till recent, Pellin, D, et al. predicted 1948 candidate sRNAs throughout *Mtb* genome using a bioinformatic pipeline based on the combination of RNA-seq data and comparative genomics [16]. In this study, we also searched for potential sRNA candidates by analyzing the transcripts mapped to IGRs. As a result, 74 and 38 intergenic transcripts were found out in log phase culture and early stationary phase culture separately (Table S9). All these transcripts were blasted against known sRNAs reported in [33,34], and no significant matches were found (E<10^-5^). This is probably attributed to the reason that we manually enriched cDNA with size mainly between 100 and 550bp before sequencing, so those transcripts with small size (<100nt) could not be detected in our results. Besides, we also blasted these intergenic transcripts in Rfam database (http://rfam.sanger.ac.uk) and found 11 hits, most of which belonged to rRNA (Table S10). But three hits from log phase intergenic transcripts belong to Cis-regulatory RNA (RF00059_TPP, RF01497_ALIL, and RF01066_6C), which implies a potential regulating function of intergenic transcripts.

In summary, we apply RNA-seq technology to explore the transcriptome of *M. marinum*. And through our research, gene expression profiles of two time points were described, with operons being predicted on genome-wide scale meantime. These results may provide insights into the molecular pathogenesis of *M. marinum*, which may shed light on the research of pathogenic *Mtb.*

## Materials and Methods

Ethics Statement: N/A

### Bacterial strains, media, and growth conditions

*M. marinum* strain M (ATCC BAA-535) was used for this study. *M. marinum* strains were grown in Middlebrook 7H9 broth (Difco) supplemented with 10% oleic acid-albumin-dextrose-catalase (OADC), 0.5% glycerol, and 0.05% Tween 80 at 32°C. Cultures were grown to log phase (OD600=0.8) and early stationary phase (12 hours after OD600 stabilized at 4.0).

### RNA isolation and removal of rRNA

Bacteria were lysed using 0.1 mm silica beads and RNA was extracted with Trizol. The RNA quality was assessed using a Nanodrop 2000 (Thermo, Fisher) and Agilent 2100 bioanalyzer. Then RNA samples were treated with DNAse and purified using RNeasy MinElute Cleanup Kit (Qiagen) before rRNA removal step. To remove rRNA, Ribo-Zero rRNA Removal Kit (Cat. No. RZPB10106, epicenter) was applied according to manufacturers’ instructions.

### RNA-seq

Construction of cDNA libraries was carried out following manufacturers’ instructions of RNA transcriptome discovery Kit (K02421-TS, Gnomegen). cDNA with range between 100bp and 550 was obtained by gel extraction followed by amplification using TruSeq PE Cluster Kit (illumina). Amplified cDNA fragments were sequenced using illumina sequencing technology (Illumina high-seq 2000). The sequencing data were submitted to the National Center for Biotechnology Information Sequence Read Archive under Accession No. SRP026200.

### Analysis of gene expression level

After adaptor trimming and quality trimming, the clean reads were mapped to the *M. marinum* transcriptome using Bowtie2 [35]. Then, we used samtools and BamIndexStats.jar to calculate the gene expression level, here RPKM value from SAM file. Gene expression difference between log and early stationary phase were obtained by MARS (MA-plot-based method with Random Sampling model), a package from DEGseq [36]. We simply defined genes with at least 2-fold change between two samples and FDR (false discovery rate) less than 0.001 as differential expressed genes.

### Quantitative real-time PCR

For quantitative real-time PCR (qRT-PCR) validation experiments, 24 genes were selected (including 10 each from up- and down-regulated genes and 4 unchanged genes according to RNA-seq data). Quantitative real-time PCR was carried out by using a TaKaRa SYBR Premix Ex Taq GC kit in a 7500 real-time PCR system (Life Technologies).

### Function category of *M. marinum* CDSs

In order to get the functional category of each *M. marinum* CDS, we started by identifying potential pairs of homologues between *M. marinum* and the well-annotated *M. tuberculosis* H37Rv CDSs. Using BLASTP search, a pair of homologues was defined by protein sequence similarity over 50%. The functional category for each pair of homologue was then referenced from Tuberculist (http://tuberculist.epfl.ch/).

### Transcripts assembly

First, all clean reads were mapped to the genome sequence using Bowtie2 [35]. Then, we did the de-novo transcriptome assembly with Velvet and Oases [37]. All de novo transcripts were then aligned to the reference genome by blat to get their genome location [38] and compared with known transcriptome annotation using cuffcompare [39]. Thus, all de novo transcripts were classified according to their positional relation with known transcripts, labeled by the evidence code provided by cuffcompare. The transcripts referred to those intergenic transcripts (class code: u).

### rRNA content calculation

We aligned all clean reads against the rRNA sequence using BLAST [40] with e-value cutoff 1e-10.

### Operon prediction and validate by RT-PCR

Operons were predicted using the following set of rules(1). Genes in an operon had the same orientation(2). Coverage of two genes was both ≥5(3). Compare the average coverage of two genes and the coverage of their IGR, the ratio should be ≤1.5(4). When two genes had no IGR, at the same time, they were in the same orientation and both coverages were ≥5, we directly calculated the ratio of their coverage, if it was ≤1.5, these two genes were considered in an operon.

RT-PCR was performed using a Takara PrimeScript RT reagent Kit with gDNA eraser. For each pair, primers were designed to amplify across the intergenic region if a contiguous transcript existed or to amplify the overlapped region (Table S11). Reactions were visualized on 1% agarose gels stained with ethidium bromide.

## Supporting Information

File S1 Figure S1, Coverage versus depth. (A) All 5452 genes were detected within two million uniquely mapped reads in log phase sample and coverage reaches a plateau afterwards despite the increasing sequencing depth. (B) One million uniquely mapped reads were able to cover all genes across whole genome in the case of stationary phase culture. Figure S2, Reproducibility of gene expression profiles by RNA-seq. A second exponential phase culture was prepared in the same procedure to evaluate the reproducibility using Spearman test. Each dot on the map stands for a single gene. The X-axis and the Y-axis correlates the expression of a single gene in two different samples. Spearman correlation coefficient = 0.867. Figure S3, Quality scores across all bases. The numbers on the x-axis indicate the position of 100 bp-long read. Values on the y-axis indicate the base quality scores. Figure S4, Comparison of correlation coefficient with RNA-seq data in *Mtb*. RNA-seq data in this study was compared with known *Mtb* transcriptome data using Spearman test [10]. The X-axis and the Y-axis correlates the expression of a single gene in different samples. Spearman correlation coefficient = 0.495 and 0.326 for log phase and early stationary phase separately. Figure S5, Correlation coefficient of each functional category comparing with RNA-seq data in *Mtb*. Correlation coefficient of each functional category was analyzed when RNA-seq data used in this study was compared with previously reported RNA-seq data in *Mtb* using Spearman Test [10]. 0 Virulence, detoxification, adaptation; 1 Lipid metabolism; 2 Information pathways; 3 Cell wall and cell processes; 5 Insertion seqs and phage; 6 PE/PPE; 7 Intermediary metabolism and respiration; 8 Unknown; 9 Regulatory proteins; 10 Conserved hypotheticals; X No homologue with H37Rv. Figure S6, RT-PCR method overview. Primers 1 and 2 were designed to amplify products across intergenic regions in the case of a contiguous mRNA transcript. Figure S7, RT-PCR results of 11 putative operons. Products of RT-PCR for 11 co-operonic gene pairs (1% agarose gel). c=cDNA template. g=genomic DNA template. r=RNA template.
(DOCX)

Table S1 **Number of reads mapped to rRNA.**
(XLSX)

Table S2 **Expression profiles of log phase and early stationary phase culture.**
(XLSX)

Table S3 **Differently expressed genes between log phase and early stationary phase culture.** (A) Genes expressed only in log phase. (B) Genes expressed only in early stationary phase.
(XLSX)

Table S4 **Median RPKM of each category.**
(XLSX)

Table S5 **qRT-PCR results.**
(XLSX)

Table S6 **Genes of each functional category.**
(XLSX)

Table S7 **Expression of genes related to ESX-1 and ESX-5.** (A) Key components of ESX-1. (B) Proteins secreted via ESX-1. (C) Key components of ESX-5. (D) Proteins secreted via ESX-5.
(XLSX)

Table S8 **Operon prediction results.** (A) Operons predicted from log phase culture. (B) Operons predicted from early stationary phase culture. (C) Overlapped operons from both cultures.
(XLSX)

Table S9 **Summary of assembly transcripts.**
(XLSX)

Table S10 **Blast results against Rfam database.**
(XLSX)

Table S11 **Primers used for operon validation.**
(XLSX)
