# Supplementary material for: Revealing of Mycobacterium marinum Transcriptome by RNA-seq
Source: PLoS One. 2013 Sep 30;8(9):e75828. doi: 10.1371/journal.pone.0075828 (PMC3786904; doi:10.1371/journal.pone.0075828)
Supplement: File S1 — Figure S1, Coverage versus depth. (A) All 5452 genes were detected within two million uniquely mapped reads in log phase sample and coverage reaches a plateau afterwards despite the increasing sequencing depth. (B) One million uniquely mapped reads were able to cover all genes across whole genome in the case of stationary phase culture. Figure S2, Reproducibility of gene expression profiles by RNA-seq. A second exponential phase culture was prepared in the same procedure to evaluate the reproducibility using Spearman test. Each dot on the map stands for a single gene. The X-axis and the Y-axis correlates the expression of a single gene in two different samples. Spearman correlation coefficient = 0.867. Figure S3, Quality scores across all bases. The numbers on the x-axis indicate the position of 100 bp-long read. Values on the y-axis indicate the base quality scores. Figure S4, Comparison of correlation coefficient with RNA-seq data in Mtb. RNA-seq data in this study was compared with known Mtb transcriptome data using Spearman test [10]. The X-axis and the Y-axis correlates the expression of a single gene in different samples. Spearman correlation coefficient = 0.495 and 0.326 for log phase and early stationary phase separately. Figure S5, Correlation coefficient of each functional category comparing with RNA-seq data in Mtb. Correlation coefficient of each functional category was analyzed when RNA-seq data used in this study was compared with previously reported RNA-seq data in Mtb using Spearman Test [10]. 0 Virulence, detoxification, adaptation; 1 Lipid metabolism; 2 Information pathways; 3 Cell wall and cell processes; 5 Insertion seqs and phage; 6 PE/PPE; 7 Intermediary metabolism and respiration; 8 Unknown; 9 Regulatory proteins; 10 Conserved hypotheticals; X No homologue with H37Rv. Figure S6, RT-PCR method overview. Primers 1 and 2 were designed to amplify products across intergenic regions in the case of a contiguous mRNA transcript. Figure S7, RT-PCR resu [file pone.0075828.s001.docx]

**Figure S1. Coverage versus depth.** (A) All 5452 genes were detected within two million uniquely mapped reads in log phase sample and coverage reaches a plateau afterwards despite the increasing sequencing depth. (B) One million uniquely mapped reads were able to cover all genes across whole genome in the case of stationary phase culture.


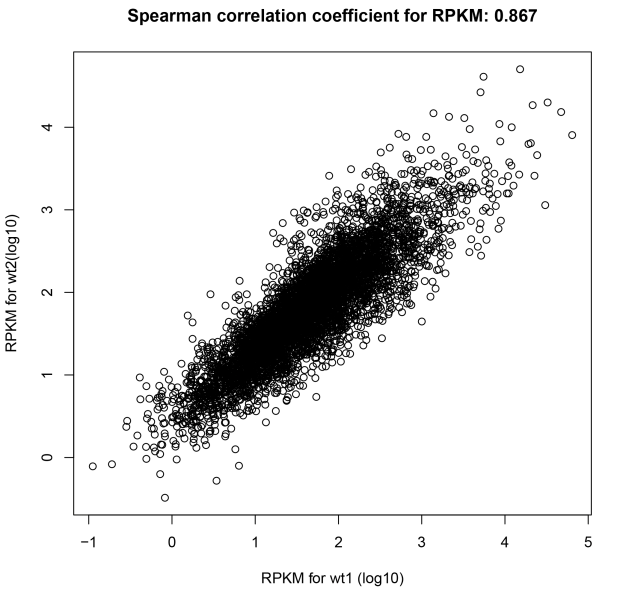


**Figure S2. Reproducibility of gene expression profiles by RNA-seq.** A second exponential phase culture was prepared in the same procedure to evaluate the reproducibility using Spearman test. Each dot on the map stands for a single gene. The X-axis and the Y-axis correlates the expression of a single gene in two different samples. Spearman correlation coefficient = 0.867.


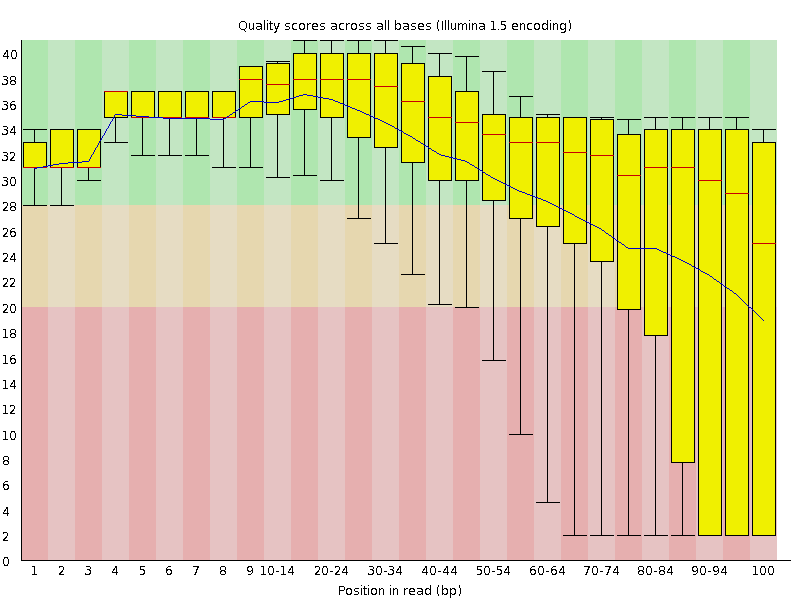


**Figure S3. Quality scores across all bases.** The numbers on the x-axis indicate the position of 100 bp-long read. Values on the y-axis indicate the base quality scores.

**Figure S4. Comparison of correlation coefficient with RNA-seq data in *Mtb*.** RNA-seq data in this study was compared with known *Mtb* transcriptome data using Spearman test [[1](#_ENREF_1)]. The X-axis and the Y-axis correlates the expression of a single gene in different samples. Spearman correlation coefficient = 0.495 and 0.326 for log phase and early stationary phase separately.

**
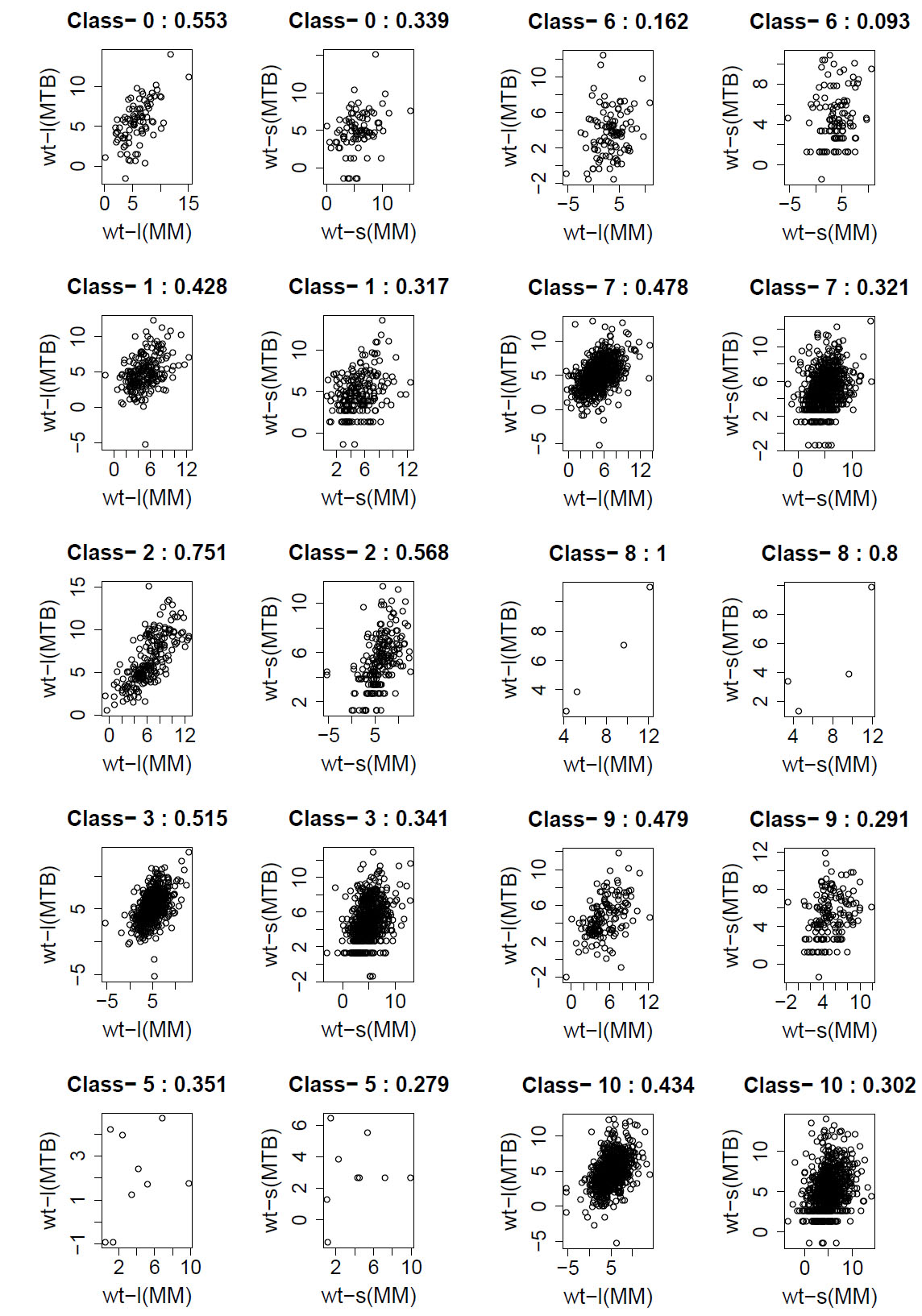
**

**Figure S5. Correlation coefficient of each functional category comparing with RNA-seq data in *Mtb*.** Correlation coefficient of each functional category was analyzed when RNA-seq data used in this study was compared with previously reported RNA-seq data in *Mtb* using Spearman Test [[1](#_ENREF_1)]. 0 Virulence, detoxification, adaptation; 1 Lipid metabolism; 2 Information pathways; 3 Cell wall and cell processes; 5 Insertion seqs and phage; 6 PE/PPE; 7 Intermediary metabolism and respiration; 8 Unknown; 9 Regulatory proteins; 10 Conserved hypotheticals; X No homologue with H37Rv.

**Figure S6. RT-PCR method overview.** Primers 1 and 2 were designed to amplify products across intergenic regions in the case of a contiguous mRNA transcript.


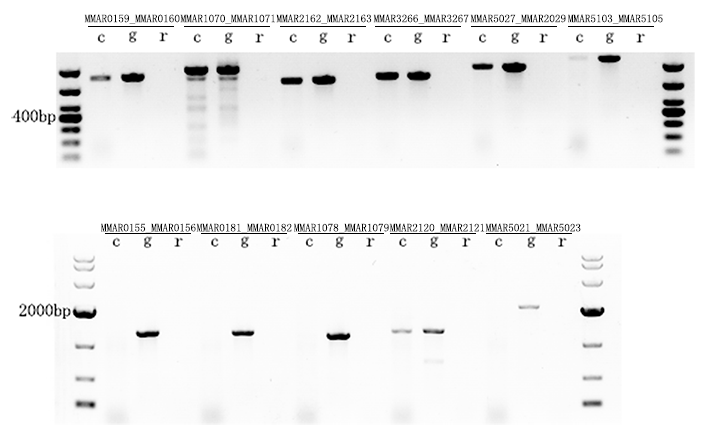


**Figure S7. RT-PCR results of 11 putative operons.** Products of RT-PCR for 11 co-operonic gene pairs (1% agarose gel). c=cDNA template. g=genomic DNA template. r=RNA template.

1. Arnvig, K.B., et al., *Sequence-based analysis uncovers an abundance of non-coding RNA in the total transcriptome of Mycobacterium tuberculosis.* PLoS Pathog, 2011. **7**(11): p. e1002342.
